# Immunotheranostic target modules for imaging and navigation of UniCAR T-cells to strike FAP-expressing cells and the tumor microenvironment

**DOI:** 10.1186/s13046-023-02912-w

**Published:** 2023-12-15

**Authors:** Liliana R. Loureiro, Lydia Hoffmann, Christin Neuber, Luise Rupp, Claudia Arndt, Alexandra Kegler, Manja Kubeil, Christoph E. Hagemeyer, Holger Stephan, Marc Schmitz, Anja Feldmann, Michael Bachmann

**Affiliations:** 1 Institute of Radiopharmaceutical Cancer Research, Helmholtz-Zentrum Dresden-Rossendorf (HZDR), Dresden, Germany; 2 Institute of Immunology, Faculty of Medicine Carl Gustav Carus, TU Dresden, Dresden, Germany; 3 Mildred Scheel Early Career Center, Faculty of Medicine Carl Gustav Carus, TU Dresden, Dresden, Germany; 4 Australian Centre for Blood Diseases, Central Clinical School, Monash University, Melbourne, Australia; 5 National Center for Tumor Diseases (NCT), Partner Site Dresden, Dresden, Germany; 6 German Cancer Consortium (DKTK), partner site Dresden, Dresden, Germany; 7 German Cancer Research Center (DKFZ), Heidelberg, Germany

**Keywords:** Cancer immunotherapy, UniCAR T-cells, Fibroblast activation protein (FAP), Tumor microenvironment (TME), 3D in vitro models, Immunotheranostic Target Modules (TMs)

## Abstract

**Background:**

Chimeric antigen receptor (CAR) T-cells are a promising approach in cancer immunotherapy, particularly for treating hematologic malignancies. Yet, their effectiveness is limited when tackling solid tumors, where immune cell infiltration and immunosuppressive tumor microenvironments (TME) are major hurdles. Fibroblast activation protein (FAP) is highly expressed on cancer-associated fibroblasts (CAFs) and various tumor cells, playing an important role in tumor growth and immunosuppression. Aiming to modulate the TME with increased clinical safety and effectiveness, we developed novel small and size-extended immunotheranostic UniCAR target modules (TMs) targeting FAP.

**Methods:**

The specific binding and functionality of the αFAP-scFv TM and the size-extended αFAP-IgG4 TM were assessed using 2D and 3D in vitro models as well as in vivo. Their specific tumor accumulation and diagnostic potential were evaluated using PET studies after functionalization with a chelator and suitable radionuclide.

**Results:**

The αFAP-scFv and -IgG4 TMs effectively and specifically redirected UniCAR T-cells using 2D, 3D, and in vivo models. Moreover, a remarkably high and specific accumulation of radiolabeled FAP-targeting TMs at the tumor site of xenograft mouse models was observed.

**Conclusions:**

These findings demonstrate that the novel αFAP TMs are promising immunotheranostic tools to foster cancer imaging and treatment, paving the way for a more convenient, individualized, and safer treatment of cancer patients.

**Graphical Abstract:**

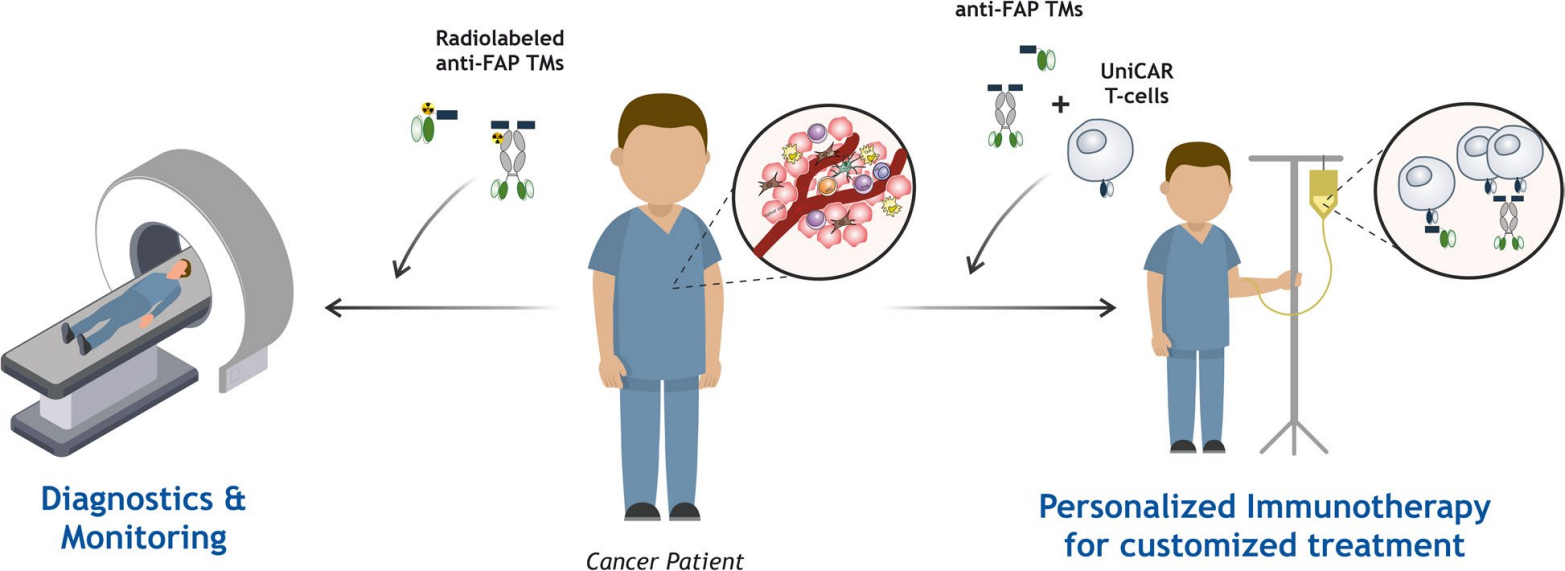

**Supplementary Information:**

The online version contains supplementary material available at 10.1186/s13046-023-02912-w.

## Background

Immunotherapeutic approaches, including CAR T-cell therapy, have revolutionized cancer treatment by leveraging immune cells to target cancerogenic cells [1]. Even though CAR T-cell therapy has demonstrated particularly remarkable success in the treatment of hematological malignancies, its efficacy when applied to solid tumors has been hampered by several hurdles, which include, for example, the immunosuppressive tumor microenvironment (TME) and antigen heterogeneity [1, 2]. Thus, it is crucial to find and target appropriate antigens as well as develop alternative and optimized CAR T-cell approaches. An example of such an alternative target antigen is the fibroblast activation protein (FAP), a cell surface protein upregulated in many cancers (over 90% of human epithelial carcinomas) and particularly highly expressed in stromal cells of the tumor microenvironment, like cancer-associated fibroblasts (CAFs) [3, 4]. FAP expression promotes tumor growth and invasion, emerging as an excellent candidate for diagnostic and therapeutic applications alongside modulation of the TME [5, 6]. Cancer therapies specifically targeting FAP are still in their early phases, with several strategies being explored, such as FAP-targeted antibodies and small molecule inhibitors, vaccine therapy, and CAR T-cell therapy (NCT03932565) [7–12]. As the TME is a complex and dynamic environment that influences the efficacy of CAR T-cell therapies, strategies to improve the outcome of such therapies may include the targeting of suitable TME-associated targets (e.g. FAP) and the optimization of CAR T-cell approaches. Alternatives to conventional CAR T-cells include the development of adapter CAR therapies, which provide greater flexibility and control in targeting cancer cells [13, 14]. The UniCAR system developed by our group is one of such modular approaches wherein an adapter molecule called target module (TM) is required and responsible for the specific bridging of UniCAR T-cells to tumor cells [15–21]. The findings from clinical studies using this approach meet the expectations related to high efficiency, safety, and controllability, aiming for its straightforward application in the treatment of both hematological and solid tumors (NCT04230265, NCT04633148) [22]. In detail, given that UniCAR T-cells express a CAR that does not recognize any surface antigen, in the absence of a TM these engineered T-cells are inert and harmless to patients. These only get activated and promote cell killing in the presence of a TM composed of a UniCAR peptide epitope (E5B9) linked to a binding moiety that specifically recognizes the target cells. Such TMs are highly versatile molecules that can be easily constructed in various formats and sizes to redirect UniCAR T-cells towards virtually any antigen [18, 23, 24]. Hence, they additionally hold great potential for diagnostic imaging applications when combined with appropriate radionuclides.

Given all the above, here we have developed novel immunotheranostic TMs with different formats and sizes for diagnostic imaging and UniCAR T-cell therapy specifically targeting human FAP. Their functionality was extensively assessed using 2D, 3D, and in vivo models, envisioning a novel combined approach to help tackle the immunosuppressive tumor microenvironment commonly found in solid cancers.

## Methods

### Cell culture

HT1080 and 3T3 cell lines were obtained from American Type Culture Collection (ATCC). The immortalized mesenchymal cell line SCP-1 was kindly provided by Dr. Martin Bornhäuser and Dr. Manja Wobus (Department of Medicine 1, University Hospital Carl Gustav Carus, TU Dresden). The fibrosarcoma cell line HT1080 was furthermore genetically modified to overexpress human FAP (hFAP) via lentiviral transduction following previously published protocols [25] and named HT1080 hFAP. To perform luciferase-based in vitro and in vivo assays, the cell lines SCP-1, HT1080 and HT1080 hFAP were additionally transduced with an open reading frame of firefly luciferase according to the protocol described by Feldmann et al. [20] and named SCP-1 Luc, HT1080 Luc and HT1080 hFAP Luc. All cell lines were cultured in DMEM supplemented with 10% FCS, 100 µg/mL penicillin/streptomycin and 1% nonessential amino acids (Sigma Aldrich) and kept at 37 °C in a humidified 5% CO_2_ atmosphere. Testing for the presence of mycoplasma by PCR was regularly performed.

### Spheroid formation

To obtain spheroids, SCP-1 cells were seeded at a density of 6 × 10^4^ or 5 × 10^3^ cells per well on either 48- or 96-well F-bottom plates coated with 1% agarose, respectively. The plates were centrifuged at 850 × *g* for 5 min and incubated for 48 h at 37 °C with 5% CO_2_. After this incubation time single spheroids were obtained and used for further assays.

### UniCAR T-cells production – T-cell isolation and genetic modification

Peripheral blood mononuclear cells (PBMCs) were isolated from buffy coats (German Red Cross, Dresden) using density gradient centrifugation with Pancoll separation solution (PanBiotech). T-cells were separated from the PBMCs via magnetic isolation using Pan T-cell Isolation Kit from Miltenyi Biotec according to the manufacturer’s instructions. After isolation, T-cells were incubated in RPMI complete medium supplemented with IL-2. Isolated T-cells were furthermore activated using T-Cell TransAct™ (Miltenyi Biotec) and lentiviral transduced with the UniCAR construct as previously described [26]. Subsequent culture and expansion were performed in TexMACS™ medium (Miltenyi Biotec) supplemented with human IL-2, IL-7 and IL-15 (Miltenyi Biotec). UniCAR T-cells were cultured in RPMI medium without cytokines for 24 h prior to experiments.

### Design, expression, purification and biochemical characterization of αFAP TMs

For the design and cloning of the αFAP TMs, the variable regions of the light (V_L_) and heavy chains (V_H_) of the humanized anti-hFAP F19 mAb [27] were fused to the UniCAR epitope E5B9. For that purpose, the lentiviral vector p6NST50 was used as detailed in previous publications [28]. The epitope E5B9 is derived from the nuclear autoantigen La/SS-B and recognized by the anti-La antibody 5B9 [29]. The epitope was selected as it is not immunogenic including in autoimmune patients either at the T-cell or B-cell level [30, 31]. It is a cryptic epitope not accessible in native La protein [32, 33]. After successful cloning, TM producing cell lines were generated by transduction of 3T3 cells with lentiviral vectors encoding for the different αFAP TMs. These were purified via His-Tag using Ni–NTA affinity chromatography, in which either Ni–NTA spin columns (Qiagen) or Ni–NTA agarose (Qiagen) on Poly-Prep® Chromatography Columns (Biorad) were used. The elution fractions were dialyzed against Phosphate buffered saline (PBS) and the concentration and purity of the TMs were determined using SDS-PAGE and Western Blot as previously reported [34, 35].

### Binding assays using flow cytometry

The binding properties and affinities of αFAP TMs were determined using flow cytometry. For that 2 × 10^5^ target cells were incubated for 1 h with varying concentrations of the TMs. Thereafter, the cells were incubated for 30 min with the anti-La mAb 5B9 and binding was finally detected after additional 30 min incubation with the goat anti-mouse Pacific Blue™ mAb (Invitrogen). Additionally, FAP expression was determined using the commercially available anti-hFAP mAb (R&D systems). All incubation steps were carried out at 4 °C and propidium iodide was used as viability marker. Stained cells were analyzed using the MACSQuant Analyzer 10 and the MACSQuantify Software from Miltenyi Biotec.

### Determination of antigen density

Antigen density on the cell surface was determined using the QIFIKIT (Quantitative Analysis Kit, Agilent) according to the manufacturer’s instructions and as previously described in detail [36]. Shortly, antigen determination was performed using the anti-hFAP mAb (R&D systems). Subsequent detection of this antibody was accomplished using the goat-anti-mouse IgG conjugated with Pacific Blue (Thermo Fisher Scientific). Analysis of the stained cells was performed using the MACSQuant Analyzer 10 and MACSQuantifiy Software (Miltenyi Biotec).

### Luciferase-based cytotoxicity assay

Killing of tumor cells by redirected UniCAR T-cells was determined using the luciferase-based cytotoxicity assay as previously described [21]. Briefly, UniCAR T-cells were incubated with luciferase-expressing monolayer target cells or spheroids at an effector to target cell (E:T) ratio of 5:1 without or with varying TM concentrations. Luminescence signals were determined after these co-cultures involving monolayer cells or spheroids were incubated for either 8 or 24 h, respectively. Data was acquired using the Nanoquant Infinite M200 Pro (Tecan).

### Cytokine-release assay

Cytokine concentration in cell-free supernatants was determined for 2D monolayer cell and 3D spheroid models. At first, 5 × 10^3^ monolayer target cells were incubated for 24 h with UniCAR T-cells in the absence or presence of αFAP TM at an E:T ratio of 5:1. For the 3D cell model the same setup was performed with the monolayer target cells being replaced by single spheroids. The concentration of each cytokine was determined using the MACSPlex Cytokine 12 Kit (Miltenyi Biotec) according to the manufacturer’s instructions. Data acquisition and analysis were performed using a MACSQuant® Analyzer and the MACSQuantify® software (Miltenyi Biotec).

### Multiplex immunohistochemistry

SCP-1 spheroids with a density of 6 × 10^4^ cells per spheroid were incubated with UniCAR T-cells at a 5:1 E:T ratio with or without TMs and incubated for 24 h. Afterwards, spheroids were fixed with 4% neutral buffered formalin (NBF) for 2 h, washed with PBS and stained with hematoxylin solution. Following another washing step, stained spheroids were embedded in Histogel™ (Fisher Scientific) on a cold metal mold. Upon dehydration and paraffin embedding, 2.5 µm thick sections were retrieved to perform further mIHC analysis. To assess the infiltration and activation status of CAR T-cells in spheroids, we employed the Opal technology (Akoya Biosciences). The staining was performed on a Ventana Discovery Ultra instrument (Ventana Medical Systems) as described previously in detail [37]. Briefly, FFPE sections were deparaffinised, rehydrated, and epitopes retrieved via heat-mediated antigen retrieval. The primary antibody was diluted in mAb diluent/block (Akoya Biosciences) according to Table 1 and added to the slides. Subsequently, the matching secondary horseradish peroxidase (HRP)-coupled OmniMap antibody (Ventana Medical Systems) and the Opal TSA fluorophore (Akoya Biosciences) were added. Bound primary and secondary antibodies were then removed by heat-mediated stripping using CC2 (Ventana Medical Systems, pH 6). Additional markers were detected by repeating the procedure from the incubation of primary antibody to the heat-mediated antibody removal (Table 1). Lastly, DAPI (Merck) was used for nuclear counterstaining. Sections were whole-scanned using the Vectra 3.0 Automated Imaging System (Akoya Biosciences) and regions of interest were defined in Phenochart™ software (Akoya Biosciences). Multispectral images (MSIs) were acquired at × 200 magnification, spectrally unmixed and exported as multi-channel tiffs in inForm software (Akoya Biosciences). Image processing for representative images was performed using ImageJ software [38].

**Table 1:** Antibodies and fluorophores used for multiplex immunohistochemistry staining of spheroids

**Antibody**	**Source**	**Clone**	**Dilution**	**Fluorophore**	**Dilution**
GrzB	Dako Agilent	Grb-7	1:200	Opal 570	1:200
CD3	Ventana Medical Systems	2GV6	Prediluted	Opal 520	1:100

### Optical imaging of experimental animals

In vivo experiments involving optical imaging of experimental mice were carried out according to the guidelines of the German Regulations for Animal Welfare. The protocol was approved by the local Ethical Committee for Animal Experiments (AZ DD24.1–5131/449/67). To assess anti-tumoral effect of UniCAR T-cells in combination with αFAP TMs, a co-injection experiment was conducted using 9-week old female NXG mice (Janvier). Each group was composed of five mice and a total of four groups were used. All the groups were injected with 0.5 × 10^6^ of HT1080 hFAP target cells. Target cells alone or in combination with UniCAR T-cells (E:T ratio of 1:1) were used as control groups. As treatment groups, mice were injected in addition with 300 pmol of either αFAP-scFv or -IgG4 TMs. Bioluminescence imaging of anesthetized mice was followed for up to 10 days using IVIS Spectrum In Vivo Imaging System (PerkinElmer) and analyzed using the Living Image Software (PerkinElmer).

### Modification of αFAP TMs with NODAGA and radiolabeling with copper-64

To modify αFAP TMs with the chelator NODAGA the first step involved the dilution of the purified αFAP TMs in modification buffer (0.1 M Na_2_B_4_O_7_*10 H_2_O, pH 9). Thereafter, p-NCS-benzyl-NODA-GA (CheMatech) was added in a molar ratio of 20:1 (chelator to TM) and incubated for 4 h at 30 °C. Non-bound p-NCS-benzyl-NODA-GA was removed by spin filtration using Millipore AmiconUltra-4 (MWCO 10,000 for αFAP-scFv TM or 50,000 for αFAP-IgG4 TM) and PBS. Once modification was completed, 1 nmol NODAGA-modified TM was added to [^64^Cu]CuCl_2_ (200 MBq, in 0.01 M HCl) and incubated for 30 min at 37 °C. The pH was adjusted to 5–6. Radiochemical yield and radiochemical purity were analyzed by radio-TLC (solid phase: iTLC-SG (Agilent), mobile phase: PBS) and radio-HPLC using a sample of the radiolabeled TMs in 2 mM aq. EDTA solution. Radiolabeled TMs were purified by spin filtration using Millipore AmiconUltra-4 (as described before) and PBS containing 2 mM EDTA and 0.0067% Dodecyl-β-D-maltoside (DDM). After that, the TMs were once again analyzed by HPLC.

The production of ^64^Cu was performed via proton irradiation of enriched ^64^Ni at a TR‐Flex cyclotron from Advanced Cyclotron Systems Inc (ACSI, Canada) and module‐assisted separation as described in detail recently [39].

### PET imaging of tumor-bearing mice

All animal experiments were carried out according to the guidelines of the German Regulations for Animal Welfare. The protocols were approved by the local Ethical Committee for Animal Experiments (DD24.1–5131/449/49). To generate tumor xenografts for positron emission tomography (PET) imaging, NMRI nude mice (Rj:NMRI-*Foxn1*^*nu/nu*^, Janvier) were subcutaneously injected with 2 × 10^6^ HT1080 and 1 × 10^6^ HT1080 hFAP cells in PBS, on the left and right hind leg, respectively. Tumor size was monitored three times a week by caliper measurements. Tumor-bearing mice were included in the imaging experiments about 7–10 days post tumor cell injection, when tumors reached a volume of at least 400 to 700 mm^3^. For small animal PET using the nanoScan PET/CT scanner (Mediso Medical Imaging Systems) tumor-bearing mice were anesthetized using desflurane, positioned and immobilized prone with their medial axis parallel to axis of the scanner. PET acquisition was started 20 s before intravenous injection of the radiotracer. Mice received 10–15 MBq radiolabeled TM delivered in 0.2 mL of 0.9% NaCl v/v through a tail vein catheter, corresponding to 60–80 pmol of the ^64^Cu-radiolabeled TM. Emission data were acquired continuously for the dedicated time points (0–60 min p.i. dynamic PET scan, 6/ 24/ 48 h p.i. static PET scans). With each PET scan, a corresponding CT image was documented and used for anatomical referencing and attenuation correction. PET data were reconstructed using Mediso Tera-Tomo™ 3D iterative reconstruction. Images were post-processed and analyzed using ROVER (ABX) and displayed as maximum intensity projections (MIPs) at the indicated time points and scaling. For PET data quantification, 3D volumes of interest (VOI) were created applying a fixed threshold (20% for tumor, heart, kidney, liver; 30% for spleen) for delineation of the organs of interest in the appropriate time frames (highest accumulation of the tracer). VOIs were transferred to all time frames for determination of standardized uptake values (SUV_mean_) and time activity curves (TACs).

### Statistical evaluation

Statistical analysis was performed using GraphPad Prism 9.0 (GraphPad Software) and statistical significance was determined using one-way ANOVA with Dunnett’s multiple comparison test or two-way ANOVA with Bonferroni’s multiple comparison. *P* values below 0.033 were considered significant as follows: *P* < 0.033 (*), *P* < 0.002 (**), *P* < 0.001 (***).

### Data availability statement

The data generated in this study are available upon request from the corresponding author.

## Results

### FAP targeting by UniCAR T-cells directed by αFAP-scFv and -IgG4 TMs

**Fig. 1:**
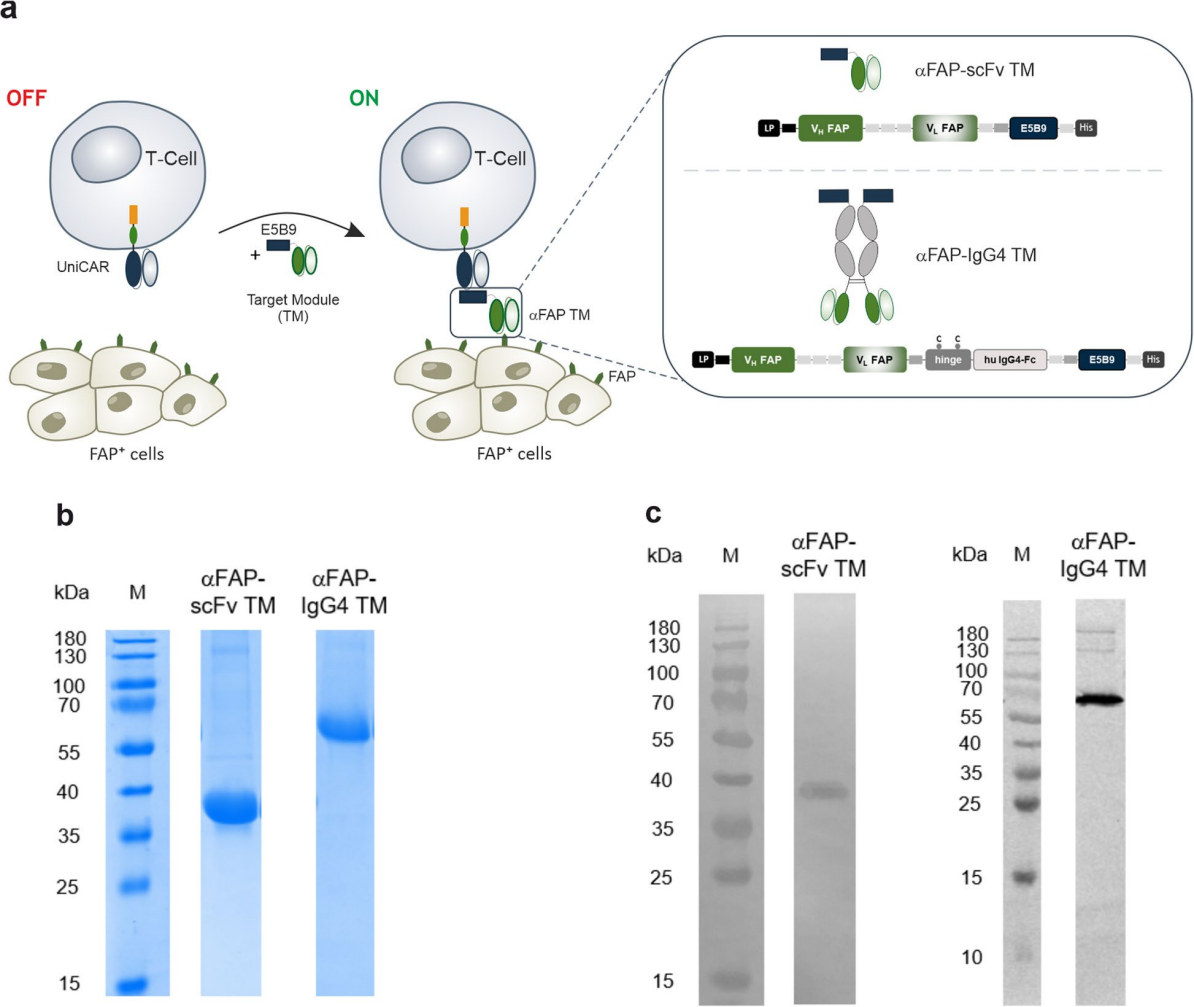
Schematic representation of the UniCAR system targeting the FAP antigen using different αFAP TM formats. **a **UniCAR T-cells consist of an extracellular single-chain fragment variable (scFv) directed to the peptide epitope E5B9, the CD28 transmembrane and intracellular signaling domains, and the CD3 zeta signaling portion. For UniCAR T-cell redirection to target FAP, an intermediary target module (TM, in this case αFAP TM) is required that is composed of a scFv that recognizes FAP and the epitope E5B9 that interacts with the UniCAR. Different formats of such TMs, e.g. scFv-based TMs (αFAP-scFv TM) and also TMs integrating the backbone of a human IgG4 (αFAP-IgG4 TM) can be combined with the UniCAR system. LP, leader peptide; V_H_, variable domain of the antibody heavy chain; V_L_, variable domain of the antibody light chain; Fc, fragment crystallizing; His, hexa-histidine tag. **b** and **c **TMs were purified via their His-tag from cell culture supernatants of producer cell lines genetically modified to permanently express the respective recombinant protein. Purified TMs were analyzed using SDS-PAGE followed by Quick Coomassie Stain (**b**) or immunoblotting followed by TM detection with the anti-La mAb 5B9 (**c**)

Considering the current interest and therapeutic potential in targeting FAP as a cancer antigen and modulator of the TME, in this work, we aimed to develop TMs specifically binding to hFAP that could be used as theranostic tools. For that, the variable domains of the light and heavy chains (V_L_ and V_H_) of a humanized monoclonal antibody (mAb) binding to hFAP [27] were linked to the E5B9 epitope, leading to the construction of a so-called αFAP-scFv TM binding on the one hand to FAP and on the other hand to UniCAR T-cells (Fig. 1a). In addition to this small-sized TM, the same variable domains were used to create an IgG4-like TM, in which they are connected to the hinge and Fc (C_H_2-C_H_3) regions of a human IgG4 molecule that are subsequently linked to the E5B9 epitope located at the C-terminus. This leads to the development of a bivalent TM named αFAP-IgG4 TM (Fig. 1a). Both TMs were further characterized and assessed for their functionality in vitro and in vivo.

After successful cloning of the above-mentioned αFAP TMs in lentiviral vectors, the murine 3T3 cell line was stably transduced to express and secrete the TMs, which in turn were purified from the cell culture medium. Purification was accomplished via His-tag using Ni–NTA affinity chromatography. Following purification, the αFAP TMs were analyzed by SDS-PAGE and immunoblotting. The theoretical molecular weights (MW) for the αFAP-scFv and -IgG4 TMs are estimated to be around 32 kDa and 112 kDa, respectively. As observed in Fig. 1b and c, main bands were obtained for SDS-PAGE and immunoblotting with mobilities according to MWs of around 37 kDa and 65 kDa for the small-sized αFAP-scFv and antibody-sized αFAP-IgG4 TMs, respectively. The MW obtained for the αFAP-IgG4 TM (lower than the predicted 112 kDa) is explained due to the reducing conditions of the SDS-PAGE, which lead to the separation of the disulfide bridges present in the native homodimer αFAP-IgG4 TM. Consequently, a band is seen at around half of the theoretical MW corresponding to the monomers of the αFAP-IgG4 TM. Moreover, the slightly increased MWs obtained from the SDS-PAGE gel in comparison to the theoretical values could be due to post-translational modifications. Overall, both αFAP TMs were successfully produced, purified, and biochemically characterized, allowing their further evaluation related to affinity and functionality.

### Development of FAP-expressing cell models and specific binding of αFAP TMs to hFAP

**Fig. 2:**
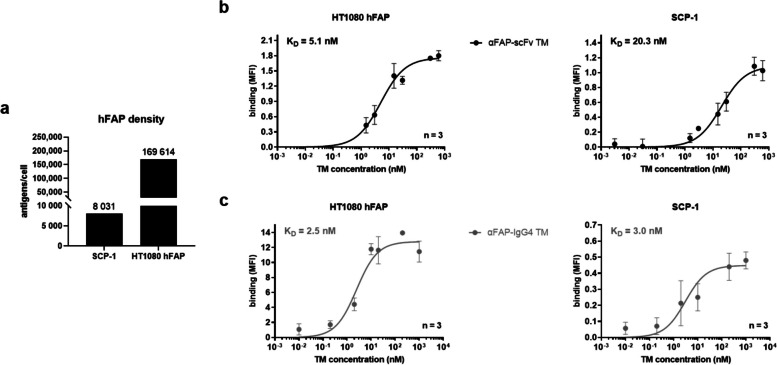
Determination of hFAP density and binding assessment of αFAP TMs using flow cytometry. **a **the number of hFAP antigens per cell was determined for SCP-1 and HT1080 hFAP cell lines using QIFIKIT. **b** and **c **Titration curves for αFAP-scFv (**b**) and -IgG4 TM (**c**) binding to HT1080 hFAP and SCP-1 cells were obtained and apparent K_D_ values were calculated. Data are plotted as MFI ± SD for three individual experiments

FAP expression is classically associated with CAFs and recognized as a marker for this type of cells. However, there are reports of mesenchymal stem cells and cancer cells expressing FAP themselves. This expression is highly variable across different types of cancer and individual tumors. Therefore, various cell lines, including glioblastoma (A172, U-87 MG), prostate (PC-3 and LNCaP), breast (MDA-MB-231), pancreatic (Panc-89), lung (A549), and colorectal (HT-29) cancer, were screened for FAP-expression on the cell surface using flow cytometry. Out of all those cell lines, FAP expression could only be detected on the surface of the mesenchymal stem cell line named SCP-1. Thus, the fibrosarcoma cell line HT1080 was genetically engineered to have an additional cell model permanently and stably expressing FAP on the cell´s surface (named HT1080 hFAP). As expected and depicted in Fig. 2a, the HT1080 hFAP cells have a considerably higher expression of hFAP on their surface, with around 170,000 FAP molecules in comparison to the naturally expressing cell line SCP-1 presenting around 8,000 FAP molecules per cell. With such cell line models established, expressing high and low FAP levels, the specific binding and binding affinity of the αFAP TMs were estimated using flow cytometry. As represented in Fig. 2b and c, the αFAP-scFv and -IgG4 TMs bind in a dose-dependent manner to both FAP-expressing cell lines with high affinity and apparent K_D_ values in the very low nanomolar range. The observed binding is specific to hFAP, as no binding could be detected on the FAP-negative parental HT1080 cells using the αFAP TMs (Supp. Figure 1a).

### UniCAR T-cells in combination with αFAP TMs promote an efficient and specific killing of FAP-expressing cells along with a specific release of pro-inflammatory cytokines using 2D models

**Fig. 3:**
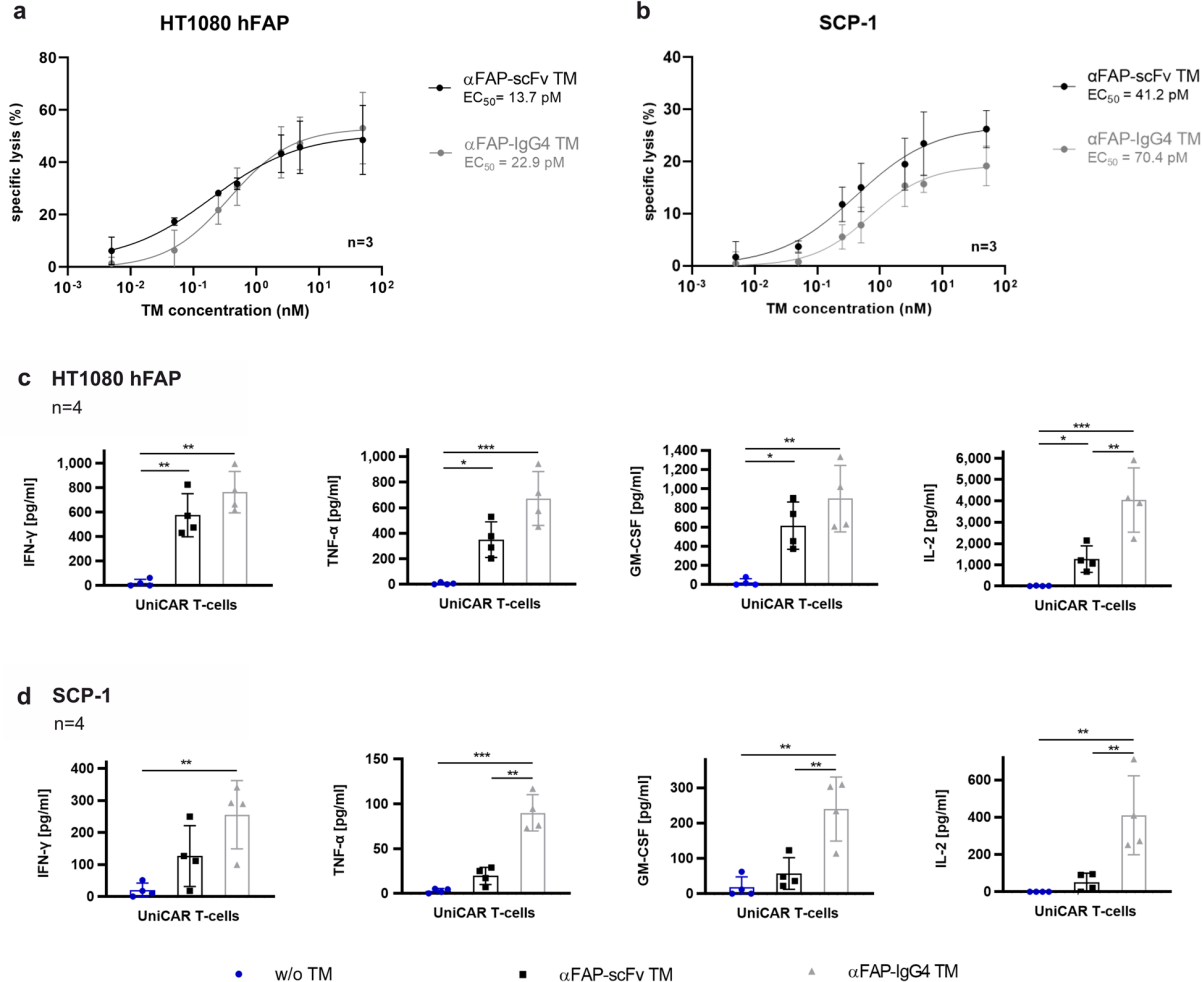
Cytotoxicity assessment, EC_50_ values determination and profile of pro-inflammatory cytokines released by UniCAR T-cells redirected by αFAP TMs. **a** and **b **titration curves assessing the specific killing of UniCAR T-cells in the presence of αFAP-scFv (black) or -IgG4 (grey) TMs in combination with HT1080 hFAP (**a**) and SCP-1 (**b**) cells were determined using luciferase-based killing assays. Dose–response curves were plotted as mean specific lysis ± SD from three individual T-cell donors and half maximal effective concentration values (EC_50_) were accordingly determined. **c** and **d **co-cultures of UniCAR T-cells with monolayer HT1080 hFAP (**c**) and SCP-1 (**d**) cells in the absence (blue) or presence of αFAP-scFv (black) and –IgG4 (grey) TMs were incubated for 24 h followed by cytokine release assessment. Scatter bar plots represent the cytokine concentrations ± SD for four individual T-cell donors. Statistical significance was determined using one-way ANOVA with Bonferroni multiple-comparison test

After corroborating the specific binding of the αFAP-scFv and -IgG4 TMs, the specific cytotoxic effect of UniCAR T-cells in combination with these TMs was further evaluated. For that, SCP-1 or HT1080 hFAP cells were co-cultured with UniCAR T-cells in the presence of different concentrations of αFAP-scFv and -IgG4 TMs. In the absence of TMs, only minimal killing (7 to 23%) was observed, and when present, these TMs were capable of redirecting UniCAR T-cells to target and eradicate FAP-expressing cells (Fig. 3a and b). Considering that the concentration of the TM plays a significant role in the controllability and effectiveness of UniCAR T-cells, the half-maximal effective concentration (EC_50_) of each one of these TMs was determined. No major variations were obtained when comparing the different αFAP TM formats, with EC_50_ values calculated to be in the low picomolar range between 14 and 70 pM. Moreover, specific eradication of FAP-expressing cells was confirmed as HT1080 (FAP-negative cells) were not killed in the presence of UniCAR T-cells and αFAP TMs (Supp. Figure 1b). Altogether, these data demonstrate that UniCAR T-cells are highly effective at killing FAP-expressing cells in the presence of αFAP TMs.

Another key mechanism of action to assess UniCAR T-cell efficacy is the release of pro-inflammatory cytokines, which influence other immune components and cells involved in the anti-tumor response. In order to assess the cytokine release profile of UniCAR T-cells, these were co-cultured with either HT1080 hFAP or SCP-1 in the absence or presence of the respective αFAP-scFv or -IgG4 TMs. Considering the specific killing results obtained previously, UniCAR T-cells were expected to secrete cytokines in a target-specific and TM-dependent manner. Indeed, the cytokine profile analysis corroborates such prospects, in which out of 12 human cytokines tested, secretion of the pro-inflammatory cytokines IFN-γ, TNF-α, IL-2, and GM-CSF was specifically obtained for the combination of UniCAR T-cells and αFAP TMs (Fig. 3c and d). In general, an increased release of cytokines was observed for the combination of UniCAR T-cells with the αFAP-IgG4 TM compared to the combination with the αFAP-scFv TM (Fig. 3c and d, grey and black bars, respectively). Additionally, negative conditions in which only UniCAR T-cells and UniCAR T-cells with αFAP-scFv or -IgG4 TMs in the absence of target cells have been included, and as expected, no release of any of the abovementioned cytokines was detected under such circumstances (Supp. Figure 2).

### Specific killing and release of pro-inflammatory cytokines by UniCAR T-cells in the presence of αFAP TMs and FAP-expressing spheroids

**Fig. 4:**
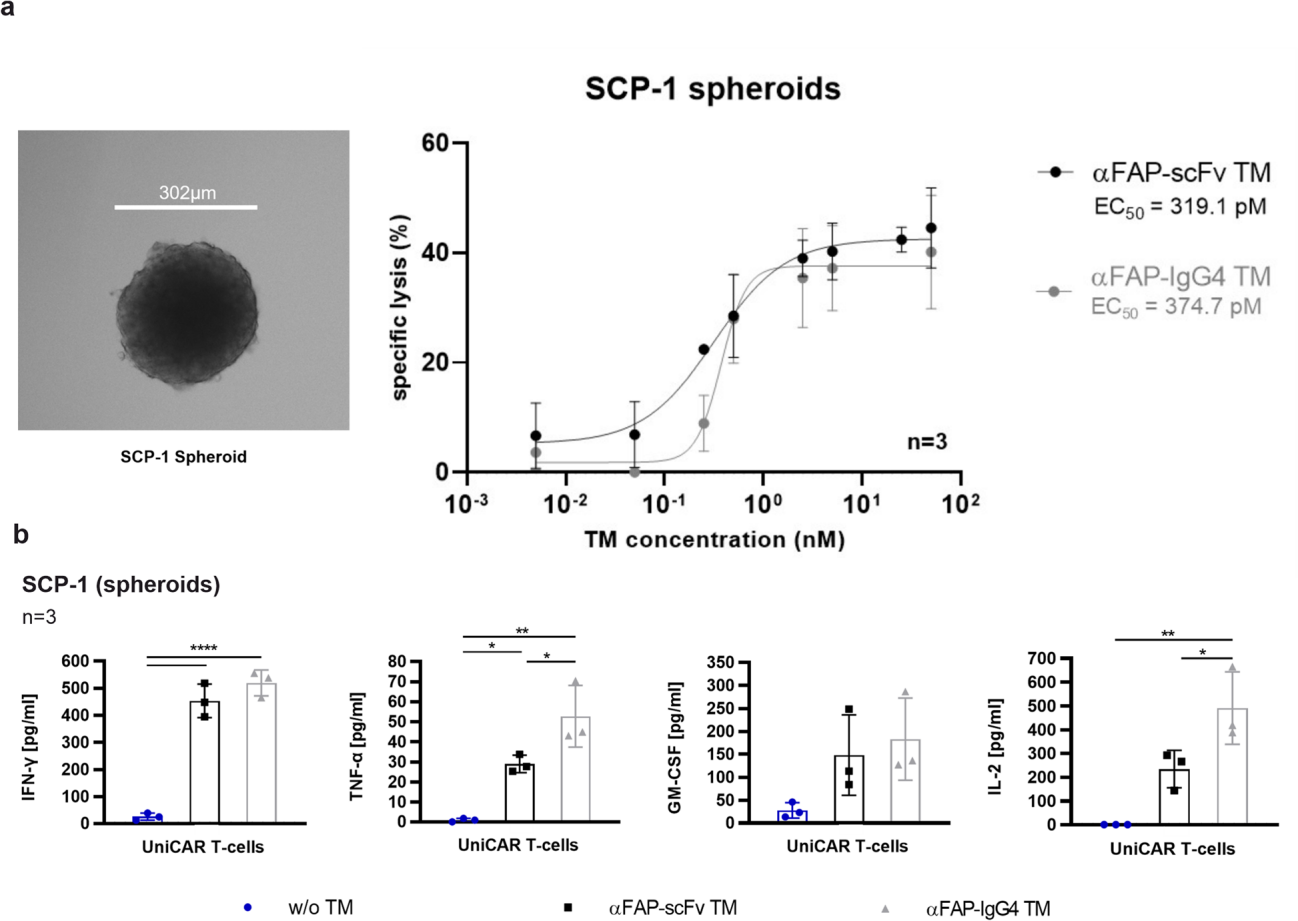
Microscopic image of a SCP-1 spheroid, cytotoxicity assessment and profile of pro-inflammatory cytokines released by UniCAR T-cells redirected by αFAP TMs to target FAP-expressing spheroids. **a** bright-field representative microscopic image of a SCP-1 spheroid obtained 48 h after cell seeding (left). Titration curves assessing the specific killing of SCP-1 spheroids by UniCAR T-cells in the presence of αFAP-scFv (black curve) or -IgG4 (grey curve) TMs in combination with SCP-1 spheroids were determined using luciferase-based killing assays. Dose–response curves and EC_50_ values were obtained and plotted as mean specific lysis ± SD from three individual T-cell donors. **b **co-cultures of UniCAR T-cells with spheroids of SCP-1 cells in the absence (blue) or presence of αFAP-scFv (black) and –IgG4 (grey) TMs were incubated for 24 h followed by cytokine release assessment. Scatter bar plots represent the cytokine concentrations ± SD for three individual T-cell donors. Statistical significance was determined using one-way ANOVA with Bonferroni multiple-comparison test

As mentioned before, FAP plays a crucial role in the modulation of the tumor microenvironment. Thus, to better mimic such complex and heterogeneous settings, 3D models of FAP-expressing cells were developed. In that way, attempts to establish spheroids from the cell lines used in this study were performed. Stable spheroids could only be obtained using SCP-1 cells (Fig. 4a). These spheroids were subsequently used to assess UniCAR T-cell-mediated killing, cytokine release, activation, and trafficking. As represented in Fig. 4a, SCP-1 spheroids were specifically eliminated by UniCAR T-cells in the presence of each one of the αFAP TMs. Concerning effectiveness and EC_50_ values using such a 3D model, both αFAP-scFv and -IgG4 TMs presented very similar values of 319 pM and 375 pM, respectively. These results demonstrate that UniCAR T-cells in the presence of specific TMs are effective in promoting cell killing not only using 2D but also more complex 3D models.

The determination of pro-inflammatory cytokine release by UniCAR T-cells co-cultured with SCP-1 spheroids in the absence or presence of αFAP TMs was performed in a similar way as for the 2D co-culture assays. Like the results obtained for the secretion of pro-inflammatory cytokines using monolayer FAP-expressing co-cultures, using 3D models we could also demonstrate that the UniCAR T-cells specifically and significantly secrete pro-inflammatory cytokines in the presence of αFAP-scFv or -IgG4 TMs (Fig. 4b). Also using such 3D conditions, an increased release of cytokines was observed for UniCAR T-cells co-cultured in the presence of the αFAP-IgG4 TM in comparison to the αFAP-scFv TM.

### UniCAR T-cells redirected by αFAP TMs specifically infiltrate FAP-expressing spheroids

**Fig. 5:**
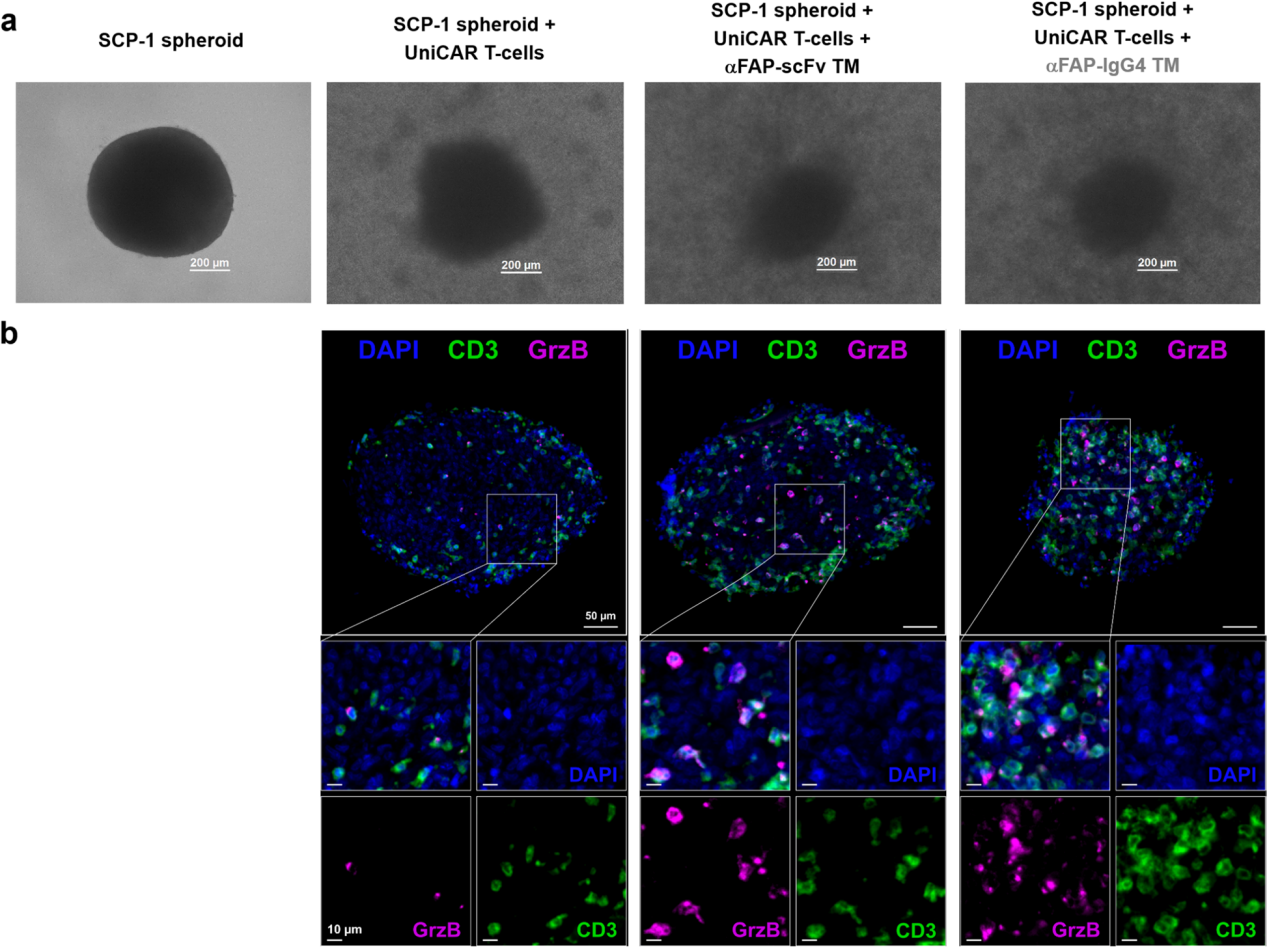
Bright-field images and multiplex immunohistochemistry (mIHC) staining of SCP-1 spheroids co-cultured with UniCAR T-cells and αFAP TMs. **a **microscopic bright-field pictures of SCP-1 spheroids alone or co-cultured for 24 h with UniCAR T-cells, UniCAR T-cells and αFAP-scFv or -IgG4 TMs. **b **representative multicolor images with magnified views of SCP-1 spheroids co-cultured for 24 h with UniCAR T-cells, UniCAR T-cells and αFAP-scFv or -IgG4 TM stained for DAPI (blue), CD3 (green) and granzyme (Grz) B (magenta). Scale bars for images in panel b indicate 50 µm in the overview images and 10 µm in the zoom-in areas

As the first evidence of targeted cell killing by UniCAR T-cells in the presence of the TMs, spheroid disintegration and size reduction were observed using bright-field microscopy (Fig. 5a). Additionally, the results obtained from the mIHC staining displayed the specific infiltration of UniCAR T-cells into the spheroid in the presence of αFAP-scFv or -IgG4 TMs. In contrast, in the images obtained for the spheroids in the presence of only UniCAR T-cells, these engineered T-cells were primarily found in the surroundings of the spheroid (Fig. 5b). The respective cytotoxic potential of UniCAR T-cells in the presence of the TMs was further corroborated, as granzyme (Grz) B expression was induced specifically under these conditions (Fig. 5b). In sum, these findings provide insight and validate the assumptions that UniCAR T-cells do not just flank the spheroids and eliminate FAP-expressing target cells from their surroundings but are also capable of infiltrating them for more successful and efficient cell killing.

### UniCAR T-cells display in vivo antitumor activity in the presence of αFAP TMs

**Fig. 6:**
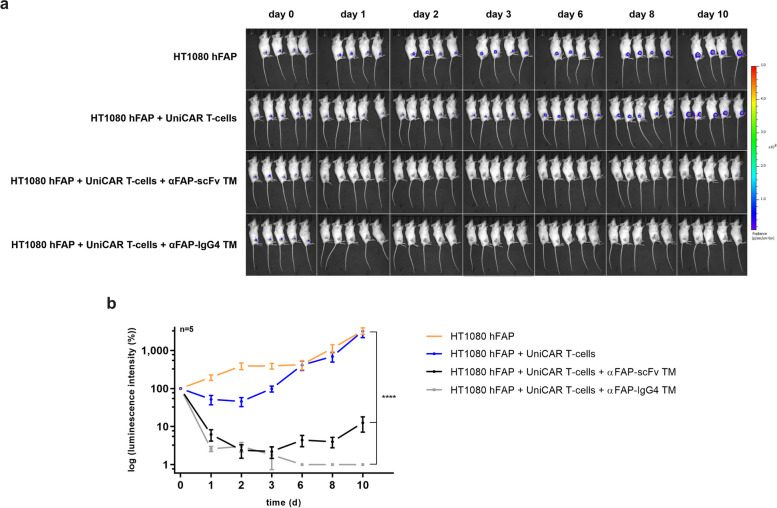
Assessment of in vivo killing of UniCAR T-cells redirected by αFAP TMs. Female NXG mice were injected with either HT1080 hFAP Luc cells (orange), HT1080 hFAP Luc cells and UniCAR T-cells (blue), or HT1080 hFAP Luc cells and UniCAR T-cells in combination with either αFAP-scFv (black) or –IgG4 TM (grey). **a **the bioluminescence signals of the HT1080 hFAP cells in all groups were monitored for 10 days. **b **based on the luminescence imaging results obtained, a quantitative analysis was performed and are represented as mean ± SD for 5 individual mice. Statistical relevance was calculated using two-way ANOVA and Dunnett multiple-comparison test with respect to the control group injected with HT1080 hFAP Luc cells alone

After gathering all the encouraging in vitro data presented before, which demonstrate the potential of using UniCAR T-cells in combination with αFAP TMs to specifically eradicate FAP-expressing tumor cells, the next step was to evaluate the immunotherapeutic effect of such a platform using a mouse model. To do so, immunodeficient mice were subcutaneously injected with HT1080 hFAP expressing firefly luciferase (HT1080 hFAP Luc) cells alone or HT1080 hFAP Luc cells and UniCAR T-cells, serving as control groups (Fig. 6). As treatment groups, mice were injected with HT1080 hFAP Luc cells mixed with UniCAR T-cells and either αFAP-scFv or -IgG4 TM. Monitoring of tumor growth was carried out by bioluminescence imaging of tumors for up to 10 days. As anticipated, tumor growth in the control groups was gradually increasing over time (Fig. 6, orange and blue curves). In contrast, a significant inhibition of tumor growth was observed for the groups treated with UniCAR T-cells in combination with αFAP-scFv or -IgG4 TMs (Fig. 6, black and grey curves). The reduction in tumor growth was more pronounced for the treatment using αFAP-IgG4 TM in comparison to using αFAP-scFv TM, achieving to some extent tumor-free mice. For the latter, a slight regrowth of tumors seems to be occurring from day 6 onward. This is most likely due to the fact that the incomplete eradication of tumor cells, together with the fast elimination of such small-sized TM, created the opportunity for remaining tumor cells to begin proliferating again.

### Remarkable in vivo tumor imaging using αFAP TMs in xenograft mouse models

**Fig. 7:**
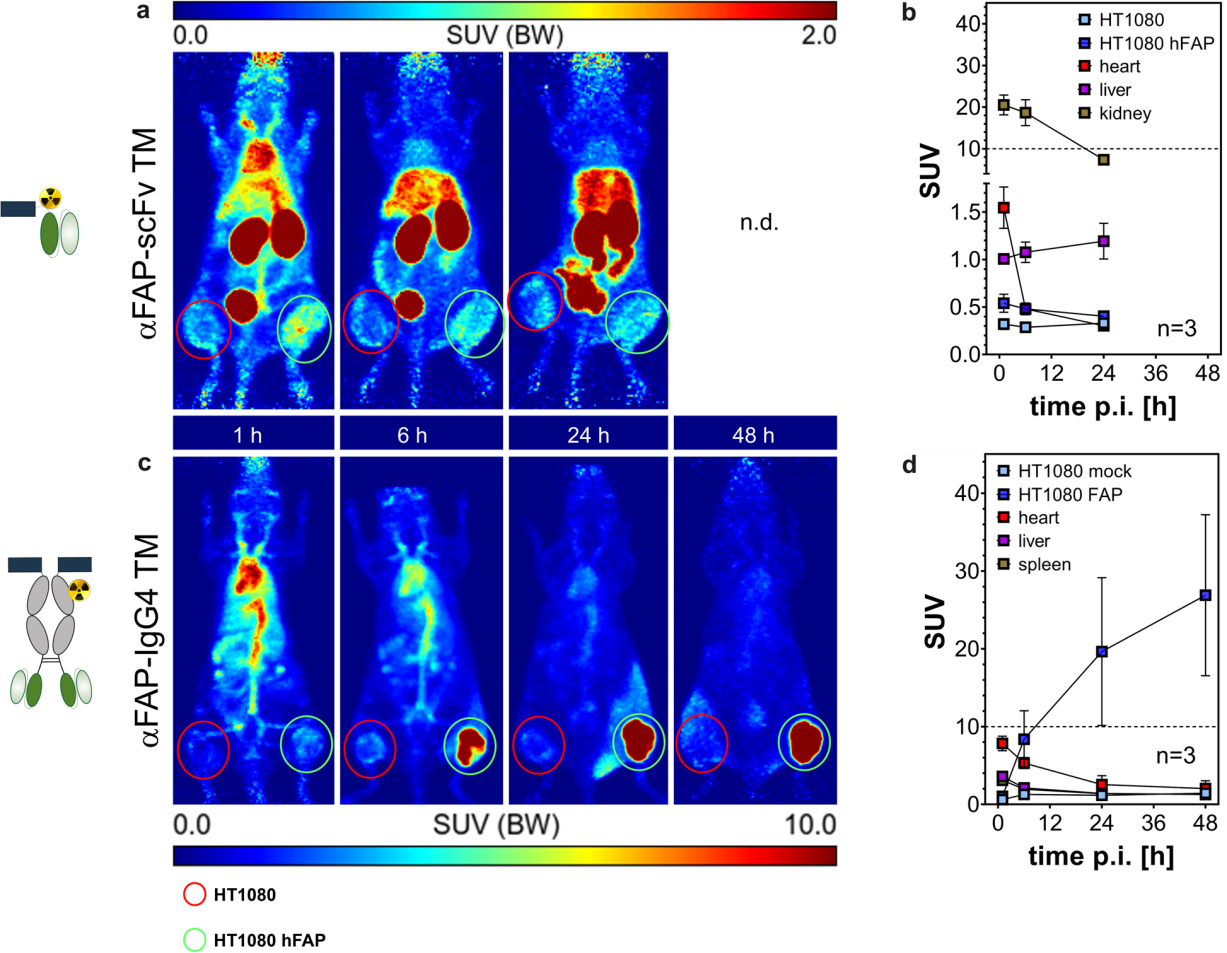
PET imaging of ^64^Cu-radiolabeled αFAP-scFv TM and -IgG4 TM in tumor xenograft mouse model. After radiolabeling with ^64^Cu, both αFAP TMs were injected intravenously in NMRI nude mice bearing HT1080 (left flank, highlighted by red circles) and HT1080 hFAP (right flank, highlighted by green circles) tumors. PET/CT imaging was performed up to 48 h post-injection (p.i.) and standardized uptake values (SUV) were calculated for the αFAP-scFv TM (**a**, **b**) and αFAP-IgG4 TM (**c**, **d**) at the different time points

In order to evaluate the diagnostic potential and specific tumor accumulation of the αFAP TMs, PET studies were conducted using mice bearing FAP-negative (HT1080) and -positive (HT1080 hFAP) tumors. Once tumor establishment was observed, the αFAP TMs were functionalized with the chelator NODAGA. After purification, the degree of conjugation was determined by MALDI-TOF MS analysis, revealing a maximum of 1 chelator per αFAP scFv TM and, on average 3 chelators per αFAP IgG4 TM, as each chelator subunit adds approximately 667 g/mol (Supp. Figure 3a). NODAGA-modified TMs were radiolabeled with copper-64 with high radiochemical purity, yields, and molar activity (around 160 GBq/µmol especially for αFAP IgG4 TM; Supp. Figure 3b-d). About 10 MBq (around 100 pmol) of radiolabeled αFAP TMs were injected intravenously, and biodistribution was analyzed by PET/CT imaging for up to 48 h p.i. and standardized uptake values (SUV) were determined for the organs of interest as well as for the HT1080 and HT1080 hFAP tumors (Fig. 7). After intravenous injection, both ^64^Cu radiolabeled αFAP TMs specifically accumulated at the site containing FAP-expressing tumor cells (Fig. 7, green circles). As expected, tumor uptake was remarkably higher for the size-extended αFAP-IgG4 TM dimer (SUV_mean_ = 26.9 ± 10.4) than for the smaller αFAP-scFv TM monomer (SUV_mean_ = 0.54 ± 0.10). The αFAP-scFv TM showed a maximum tumor uptake already after 1 h p.i., whereas the αFAP-IgG4 TM seems to start accumulating at the FAP-positive tumor site at around 6 h p.i., reaching its maximum tumor accumulation at around 48 h p.i. Besides its increased tumor uptake and persistence, the αFAP-IgG4 TM demonstrated prolonged blood circulation and delayed clearance due to its extended molecular size. Along with this, the accumulation of this TM in the liver or kidneys could barely be detected. On the contrary, and given the considerably higher kidney and liver retention, the smaller αFAP-scFv TM was rapidly excreted via renal and hepatobiliary pathways. To confirm specific TM accumulation at the HT1080 hFAP tumor site, after the PET studies, both tumors were collected, and FAP expression on the tumor lysates was determined using immunoblotting. The results confirm that FAP expression was restricted in the HT1080 hFAP tumors and absent in HT1080 tumor cells (Supp. Figure 4). Overall, the results obtained demonstrate that αFAP TMs are suitable for PET imaging of FAP-expressing tumors. Notably, the suitability of the size-extended αFAP-IgG4 TM dimer as a diagnostic PET tracer is particularly highlighted by its notably high and specific accumulation at the tumor site, with basically no accumulation in any other organs.

## Discussion

CAR T-cells are undoubtedly an immunotherapeutic approach with promising clinical applications in the treatment of several types of cancer. Despite the success treating certain blood cancers, such as acute lymphoblastic leukemia (ALL) and non-Hodgkin lymphoma (NHL), various challenges hamper the effectiveness and application of CAR T-cell therapy in solid tumors [1, 2, 40]. Within those challenges, the complex and immunosuppressive TME plays a key role, in which many non-cancerous cells, such as immune cells, fibroblasts as well as extracellular matrix (ECM) components and cytokines, mutually support tumor proliferation and invasion [1]. Among others, strategies that could be used to modulate the TME involve, for example, the targeting of stromal cells like CAFs. These are reported to overexpress FAP and are responsible for supporting tumor progression, modulating the immune response within the TME, and inducing radioresistance [41–44]. In addition, FAP has been reported to be overexpressed in a variety of cancers, such as lung, breast, and colorectal, making it an attractive antigen for the simultaneous targeting of tumor cells and respective CAFs present within the TME [45–47]. Given our expertise working with modular CAR T-cell therapies and engineered antibody derivatives, we designed TMs against FAP to be exploited as theranostic tools with potential to modulate the immunosuppressive TME. To pursue this aim, two αFAP TMs were developed, which differ in their structure and size. Having these TM formats coupled with appropriate radionuclides, we expect to obtain different pharmacokinetics that allow improved imaging and diagnostics of FAP-expressing tumors. Likewise, TMs with such pharmacokinetic variances in combination with UniCAR T-cells will allow a customized treatment based on the patient´s needs, in which UniCAR T-cell activity can be easily managed by TM dosing and interplay between the different formats, for example, in case of side effects or based on tumor burden.

To assess the functionality of the novel αFAP TMs, two cell models were used: the naturally expressing FAP cell line SCP-1 and the engineered cell line HT1080 hFAP, expressing low and high levels of hFAP on the cell surface, respectively. Binding studies using these cell lines demonstrate that both αFAP-scFv and -IgG4 TMs specifically bind with high affinity to hFAP. Similar results were obtained concerning the killing of FAP-expressing cells, in which no major differences were observed in the cytotoxic potential of UniCAR T-cells redirected by αFAP-scFv or -IgG4 TMs targeting both naturally expressing and overexpressing FAP cell lines. Such results may suggest that the affinity and efficacy of the mAb used to originate the αFAP scFv fragments are high enough and that the increased valency of the αFAP-IgG4 TM does not seem to have a substantial impact on binding or killing for the cell models used. The variance in antigen density on the surface of the natively expressing (SCP-1) in comparison to the hFAP-engineered (HT1080 hFAP) cell line had, as expected, an impact on maximum specific killing abilities but not on the effective killing concentrations when comparing the two αFAP TMs. Together with such indications, it is of high relevance to evaluate the secretion of pro-inflammatory cytokines by UniCAR T-cells, as these signaling molecules play a key role in the immune response against tumors. A controlled release is crucial to avoid potential severe complications in patients. The cytokine release data demonstrate the specific release of pro-inflammatory cytokines by UniCAR T-cells in the presence of αFAP TMs and FAP-expressing cells. This production seems to be increased in the presence of αFAP-IgG4 TMs compared to αFAP-scFv suggesting that the bivalency in this case might be beneficial to overcome the threshold for cytokine induction. Overall, these data corroborate the improved safety and efficacy of the UniCAR platform.

In vitro models of the TME are important tools in the development of new cancer immunotherapies, allowing a deeper understanding of the interactions between the different cell types and the surrounding environment. Yet, it´s not that straightforward to mimic and study it using only conventional 2D in vitro models. To address such obstacles, 3D in vitro models have been widely used and optimized [48]. Among others, spheroids have proven to better mimic the in vivo conditions of the TME in comparison to 2D cell culture models [48]. For this reason, we developed spheroid models to assess the suitability of FAP targeting using αFAP TMs and UniCAR T-cells. With regard to killing and cytokine secretion, the results obtained using FAP-expressing spheroids substantiate the results obtained using the 2D models, in which specific cell killing and pro-inflammatory cytokine release were observed explicitly in the presence of the αFAP-scFv and αFAP-IgG4 TMs. The half-maximal effective concentrations of the αFAP TMs and the cytokine secretion values of redirected UniCAR T-cells achieved when targeting spheroids were generally lower compared to the 2D cell monolayer setting. These findings can be explained by the increased complexity and density of a spheroid in comparison to monolayered models, which leads to a slight delay in cytokine secretion and increased amounts of TMs are required to obtain effective results. Nonetheless, the UniCAR T-cells redirected by αFAP TMs have proven to effectively and specifically eradicate FAP spheroids. Moreover, we subsequently show that the UniCAR T-cells are predominantly located in the surroundings of the spheroids, patiently waiting to be redirected and activated in the presence of the αFAP TMs. And indeed, mIHC imaging displays the activation and infiltration of the UniCAR T-cells into the spheroids and their consequent disintegration exclusively in the presence of these TMs. Such models highlight the unique strength of the UniCAR platform and set the stage to establish more complex models encompassing, in addition, e.g. the interaction with CAFs and immune cells to better address the suitability of our system in the targeting and modulation of the TME.

Last but not least, the immunotheranostic potential of the αFAP TMs was evaluated using mouse models. Exploiting xenograft mouse models, we observed that tumor growth was significantly reduced exclusively in the presence of UniCAR T-cells and αFAP-scFv or αFAP-IgG4 TMs. A slight tumor regrowth was observed in the mice treated with UniCAR T-cells and αFAP-scFv TM in comparison to the group treated with αFAP-IgG4 TM. Such an effect can be explained by the reduced size of the αFAP-scFv TM, which leads to its rapid elimination from the body and consequent proliferation of the remaining tumor cells. A constant or repeated supply of αFAP-scFv TM would be needed to maintain such tumor regression observed right after TM injection. On the other hand, the bivalent αFAP-IgG4 TM has extended blood circulation and longer retention time at the tumor site, resulting in a prolonged and more efficient inhibition of tumor growth over time without the need for continuous TM infusion. These observations sustain previously reported work in which a precise combination of small-sized and extended half-life TMs with UniCAR T-cells is relevant in the management of a more convenient treatment for cancer patients during therapy [23]. Particularly when it comes to addressing the TME, having both TM formats may be of high value in designing fitting strategies according to the antigens and cells to target within the immunosuppressive TME. We envision that in a clinical scenario, the small-sized αFAP-scFv TM could be used to launch an initial strike to destabilize the TME since the diffusion of this TM should be faster through the TME, promoting also a faster recruitment of the UniCAR T-cells and consequent activation and killing of FAP-expressing cells. Additionally, the small-sized αFAP-scFv TM provides the possibility to rapidly switch off unwanted UniCAR T-cell reactions at an early treatment phase. This initial approach would alleviate immunosuppression, helping to improve even more the penetration and functionality of UniCAR T-cells for a subsequent treatment using αFAP-IgG4 TMs. Along with this TM´s extended half-life and prolonged accumulation at the tumor site, we would expect a persistent activation and eradication of target cells, substantially reducing the tumor load or even completely eradicating it.

The herein presented preclinical studies demonstrate proof of immunotherapeutic functionality of the UniCAR system for the targeting of FAP. To further evaluate its clinical potential, more complex models may be of interest to better mimic the TME, such as 3D in vitro co-culture assays using different immune cell types present in the TME, patient-derived organoids, along with suitable mouse models.

Besides immunotherapy, the potential of such TMs to be used for diagnostic as well as therapeutic monitoring was assessed using PET imaging. The data acquired reveals a particularly outstanding specificity and high accumulation at the tumor site using the αFAP-IgG4 TM. Additionally, labeling of αFAP TMs with therapeutic radionuclides may be of high interest for endoradiotherapy approaches and will be investigated in prospective studies. Another interesting strategy that could be followed would be combining targeted radionuclide therapy (endoradiotherapy) with UniCAR immunotherapy. In this case, the αFAP TMs would be coupled to suitable therapeutic radionuclides to help overcome the TME barriers as a first approach, enhancing the trafficking and effectiveness of subsequent immunotherapy using unmodified αFAP TMs and UniCAR T-cells. With such striking results, our FAP targeting UniCAR platform has the potential to be used alone or combined with other approaches for cancer therapy and diagnostic imaging, representing an attractive theranostic targeting strategy.

## Conclusions

Existing cancer therapies specifically targeting FAP are currently being evaluated for different types of cancer, and we believe that such a modular strategy like the UniCAR system may bring a considerable advantage based on the results obtained in this work, with increasing safety and efficacy along with modulation of the TME. In addition, the versatility of the αFAP TMs and proven specific accumulation at FAP-expressing sites pave the way to a new era of cancer diagnostics and therapy for solid tumors and their immunosuppressive TME, in which one molecule could be used for diagnosis, tumor monitoring, and treatment involving both radiotherapeutic and immunotherapeutic approaches.

### Supplementary Information

**Additional file 1:** **Supp. Fig.** **1.** – Binding and killing assessment of aFAP TMs to FAP-negative cells. As a control for specific binding and killing, flow cytometry (a) and luciferase-based killing assays (b) using HT1080 cells (FAP-negative cells) in the presence of 50nM aFAP TMs were performed, respectively. One representative measurement or donor is shown.** Supp. Fig**. **2.** – Profile of pro-inflammatory cytokines released by UniCAR T-cells in the presence of aFAP TMs and absence of target cells. UniCAR T-cells alone or in the presence of 50nM aFAP-scFv (square) or–IgG4 (triangle) TMs were incubated for 24h followed by cytokine release assessment. Scatter bar plots represent the cytokine concentrations ± SD for four individual T-cell donors, **Supp. Fig. 3.** – MALDI TOF MS, Radio-SDS-PAGE, and Radio-HPLC of radiolabeled aFAP TMs. a, TMs were conjugated with the Cu-chelator NODAGA and degree of conjugation was determined by MALDI-TOF MS. b and c, radiolabeled aFAP TMs were analyzed using SDS-PAGE followed by Radioluminography (b) and Coomassie Brilliant Blue staining (c). d. radiochemical purity was analyzed using Radio-HPLC. **Supp. Fig. 4.** – Immunoblotting of tumor lysates from mouse models. Cell lysates obtained from tumors extracted from the mice were stained for FAP expression using immunoblotting. GAPDH expression was used as control.

## Data Availability

Data confirming the results of this study are presented in the manuscript and are available from the corresponding author upon reasonable request.

## Abbreviations

ALL: Acute lymphoblastic leukemia
CAFs: Cancer-associated fibroblasts
CAR: Chimeric antigen receptor
C_H_2: Constant domain 2 of the heavy chain of a monoclonal antibody
C_H_3: Constant domain 3 of the heavy chain 3 of a monoclonal antibody
CT: Computed tomography
DDM: Dodecyl-β-D-maltoside
DMEM: Dulbecco’s modified Eagle’s medium
EC_50_: Half maximal effective concentration
EDTA: Ethylenediamine tetraacetic acid
EGFP: Enhanced green fluorescent protein
ELISA: Enzyme-linked immunosorbent assay
E:T: Effector to target cells
FAP: Fibroblast activation protein
FBS: Fetal bovine serum
HRP: Horseradish peroxidase
IgG4: Immunoglobulin 4
K_D_: Equilibrium dissociation constant
Luc: Firefly luciferase; mAb: monoclonal antibody
MALDI-TOF MS: MALDI coupled to time-of-flight mass spectrometry
MDSCs: Myeloid derived suppressor cells
MFI: Median fluorescence intensities
MIP: Maximum intensity projection
MSI: Multispectral images
MW: Molecular weight
NBF: Neutral buffered formalin
NHL: Non-Hodgkin lymphoma
PBMCs: Peripheral blood mononuclear cells
PBS: Phosphate buffered saline
PET: Positron emission tomography
scFv: Single-chain variable fragment
SD: Standard deviation
SDS-PAGE: Sodium dodecyl sulfate polyacrylamide gel electrophoresis
HPLC: High-performance liquid chromatography
SUV: Standard uptake values
TAC: Time activity curve
TLC: Thin layer chromatography
TM: Target module
TME: Tumor microenvironment
UniCAR: Universal CAR
V_H_: Variable domain of the heavy chain of a monoclonal antibody
V_L_: Variable domain of the light chain of a monoclonal antibody
VOI: Volume of interest
WB: Western blot
