# Supplementary material for: Immunotheranostic target modules for imaging and navigation of UniCAR T-cells to strike FAP-expressing cells and the tumor microenvironment
Source: J Exp Clin Cancer Res. 2023 Dec 15;42:341. doi: 10.1186/s13046-023-02912-w (PMC10722841; doi:10.1186/s13046-023-02912-w)
Supplement: Supplementary file 1 — Additional file 1: Supp. Fig. 1. – Binding and killing assessment of aFAP TMs to FAP-negative cells. As a control for specific binding and killing, flow cytometry (a) and luciferase-based killing assays (b) using HT1080 cells (FAP-negative cells) in the presence of 50nM aFAP TMs were performed, respectively. One representative measurement or donor is shown. Supp. Fig. 2. – Profile of pro-inflammatory cytokines released by UniCAR T-cells in the presence of aFAP TMs and absence of target cells. UniCAR T-cells alone or in the presence of 50nM aFAP-scFv (square) or–IgG4 (triangle) TMs were incubated for 24h followed by cytokine release assessment. Scatter bar plots represent the cytokine concentrations ± SD for four individual T-cell donors, Supp. Fig. 3. – MALDI TOF MS, Radio-SDS-PAGE, and Radio-HPLC of radiolabeled aFAP TMs. a, TMs were conjugated with the Cu-chelator NODAGA and degree of conjugation was determined by MALDI-TOF MS. b and c, radiolabeled aFAP TMs were analyzed using SDS-PAGE followed by Radioluminography (b) and Coomassie Brilliant Blue staining (c). d. radiochemical purity was analyzed using Radio-HPLC. Supp. Fig. 4. – Immunoblotting of tumor lysates from mouse models. Cell lysates obtained from tumors extracted from the mice were stained for FAP expression using immunoblotting. GAPDH expression was used as control. [file 13046_2023_2912_MOESM1_ESM.pdf]

Immunotheranostic target modules for imaging and navigation of UniCAR T-cells to strike FAP-expressing cells and the tumor microenvironment

Liliana R. Loureiro[1,†,*], Lydia Hoffmann[1,†], Christin Neuber[1], Luise Rupp[2], Claudia Arndt[1,3], Alexandra Kegler[1], Manja Kubeil[1], Christoph E. Hagemeyer[4], Holger Stephan[1], Marc Schmitz[2,5,6], Anja Feldmann[1,6,7,*], Michael Bachmann[1,6,7,*]

SUPPLEMENTARY INFORMATION

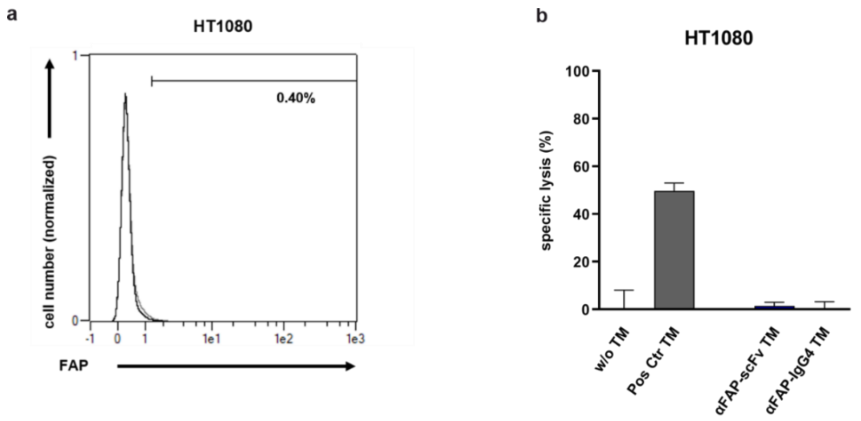

Supp. Fig. 1 – Binding and killing assessment of αFAP TMs to FAP-negative cells. As a control for specific binding and killing, flow cytometry (**a**) and luciferase-based killing assays (**b**) using HT1080 cells (FAP-negative cells) in the presence of 50nM αFAP TMs were performed, respectively. One representative measurement or donor is shown.

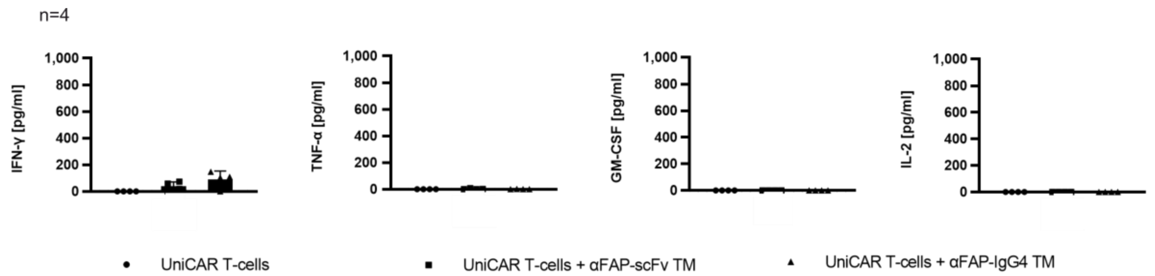

Supp. Fig. 2 – Profile of pro-inflammatory cytokines released by UniCAR T-cells in the presence of αFAP TMs and absence of target cells. UniCAR T-cells alone or in the presence of 50nM αFAP-scFv (square) or –IgG4 (triangle) TMs were incubated for 24h followed by cytokine release assessment. Scatter bar plots represent the cytokine concentrations ± SD for four individual T-cell donors.

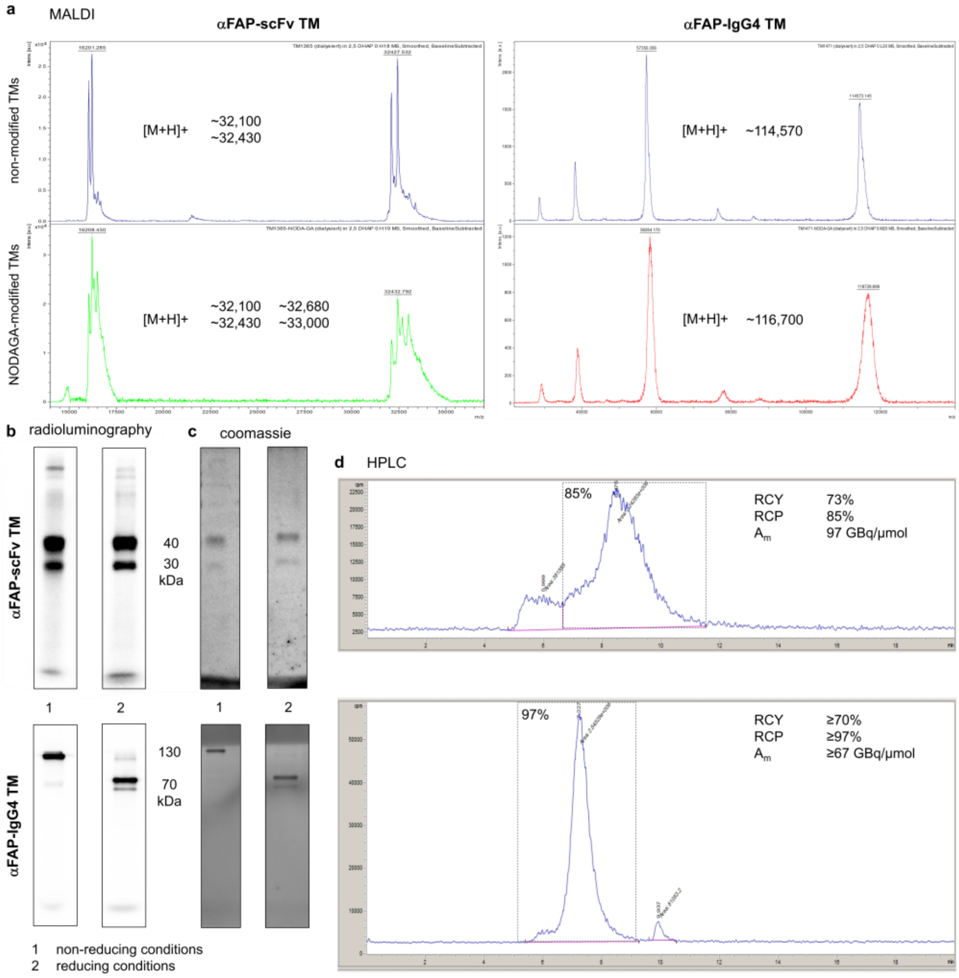

Supp. Fig. 3 – MALDI TOF MS, Radio-SDS-PAGE, and Radio-HPLC of radiolabeled αFAP TMs. **a**, TMs were conjugated with the Cu-chelator NODAGA and degree of conjugation was determined by MALDI-TOF MS. **b and c**, radiolabeled αFAP TMs were analyzed using SDS-PAGE followed by Radioluminography (**b**) and Coomassie Brilliant Blue staining (**c**). **d**, radiochemical purity was analyzed using Radio-HPLC.

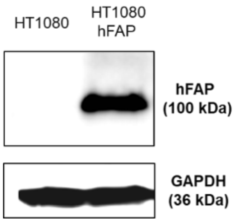

Supp. Fig. 4 – Immunoblotting of tumor lysates from mouse models. Cell lysates obtained from tumors extracted from the mice were stained for FAP expression using immunoblotting. GAPDH expression was used as control.

SUPPLEMENTARY MATERIALS AND METHODS

Matrix-assisted laser desorption ionisation time-of-flight (MALDI-TOF) mass spectrometry

MALDI-TOF mass spectrometry was performed as described elsewhere (S1). In brief, 2,5-Dihydroxyactetophenone (2,5-DHAP,Bruker Daltonik) was used as matrix for MALDI-TOF MS. An

aliquot of the protein sample was mixed with 2 mL of 2% TFA solution. After addition of 2 mL of matrix solution, the mixture was pipetted up and down until the crystallization started and the solution became cloudy. Finally, 0.5 mL of the crystal suspension was spotted onto the ground steel target plate and the droplet was air-dried completely at room temperature. Spectra were acquired with an autoflex II TOF/TOF (Bruker Daltonik) in positive linear mode in combination with the flexControl software (Version 3.3, Bruker Daltonik) and analyzed with the flexAnalysis software (Version 3.3, Bruker Daltonik). Theoretical molecular weights were calculated using the Compute pI/Mw tool on the ExPASy Server.

Radio-SDS-PAGE of radiolabeled αFAP TMs

Radiolabeled TMs were diluted in Laemmli buffer either without or with 2-mercaptoethanol (5 mM) to analyze under non-reducing or reducing conditions, respectively. Samples were run on a SDS gel along with the PageRuler™ Plus Prestained Protein Ladder (Thermo Fisher Scientific). Radioactive bands were visualized using the Amersham Typhoon 5 Biomolecular Imager (Cytiva). Afterwards, SDS gels were stained with Quick Coomassie® Stain (Serva Electrophoresis). Protein bands were again visualized using the Amersham Typhoon 5 Biomolecular Imager (Cytiva).

Characterization of HT1080 tumor xenografts by immunoblotting

To confirm expression of hFAP also persists during tumor growth, tumors were resected immediately after mice were euthanized and frozen using cold (-20°C) 2-Methylbutane (Sigma Aldrich). Preparation of cell lysates from the resected tumors and immunoblotting analysis were performed as previously described elsewhere (S2). In brief, proteins were separated in SDS-PAGE and transferred to a PVDF membrane (Fisher Scientific) using the Trans-Blot® Turbo™ Transfer System (Bio-Rad). After blocking, the membrane was incubated with rabbit anti-human FAP mAb (Abcam) for 2 h at room temperature followed by overnight incubation at 4°C. Thereafter, washings were performed followed by incubation with peroxidase goat anti-rabbit IgG (Sigma Aldrich). Protein bands were detected with Super Signal West Pico and Femto Chemiluminescent Substrate (Thermo Fisher Scientific) and the chemiluminescence imager CELVIN® S 420 (Biostep). As loading control, membranes were re-analyzed with mouse anti-GAPDH (Sigma) and peroxidase coupled goat anti-mouse IgG (Sigma Aldrich).

REFERENCES

S1. Singh G, Zarschler K, Hunoldt S, Martínez IIS, Ruehl CL, et al. Versatile Bispidine-Based Bifunctional Chelators for $^{64}Cu^{II}$ -Labelling of Biomolecules. Chemistry 2020;26:1989-2001.

S2. Mamat C, Mosch B, Neuber C, Köckerling M, Bergmann R, Pietzsch J. Fluorine-18 Radiolabeling and Radiopharmacological Characterization of a Benzodioxolylpyrimidine-based Radiotracer Targeting the Receptor Tyrosine Kinase EphB4. Chem Med Chem; 2012;7:1991–2003.
